# Metabolite medicine offers a path beyond lists of metabolites

**DOI:** 10.1038/s42004-021-00551-w

**Published:** 2021-08-05

**Authors:** Shira Shaham-Niv, Sigal Rencus-Lazar, Ehud Gazit

**Affiliations:** 1 BLAVATNIK CENTER for Drug Discovery, Tel Aviv University, Tel Aviv, Israel; 2 The Shmunis School of Biomedicine and Cancer Research, The George S. Wise Faculty of Life Sciences, Tel Aviv University, Tel Aviv, Israel

**Keywords:** Metabolomics, Metabolic pathways

## Abstract

Aimed to decipher the levels of metabolites, metabolomics can now advance to unraveling their functionalities in various contexts. Here, the authors present the metabolite medicine concept, integrating classical metabolomics methods with advanced computational and structural tools to facilitate functional studies.

## The metabolomics era

The ability to accurately determine the composition and concentrations of large sets of metabolites at varying physiological and pathological states had transformed our understanding of biological processes. The roles played by metabolites are highly diverse, including, among others, structural functions, regulation of signal transduction, feedback mechanisms and generation of photonic systems *via* supramolecular self-assembly. The levels of metabolites are strictly regulated in metabolomic networks^1^, giving rise to a dynamically-stable metabolome homeostasis, termed metabolostasis. Consistently, the abrogation of these functions is often associated with pathological outcomes. Thus, inborn error of metabolism (IEM) disorders arise from congenital mutations in genes encoding metabolic enzymes, often resulting in aberrant accumulation of various metabolites. Recently, the accumulated metabolites in several IEM conditions have been suggested to self-assemble into ordered cytotoxic structures, which may account for the pathological phenotypes^2–4^. Similarly, crystallization of metabolites, such as uric acid and calcium oxalate, is known to be associated with various pathologies, including gout and kidney stones, respectively^5,6^. The involvement of diverse metabolites in different types of cancer is also beginning to unfold, designating these molecules as oncometabolites^7^. Other conditions, such as neurodegenerative and cardiovascular diseases, have also been shown to involve de-regulation of metabolite levels. However, in spite of the numerous indications for the important role metabolites play in physiology and pathology, in most cases, their exact functions are still unknown, emphasizing the need to decipher the mechanisms underlying these conditions. Such an understanding will allow to identify whether a given metabolite plays a causative role in a specific pathology, or whether its aberrant levels are merely a side effect.

Following the advancements achieved through genomics and proteomics, the realization of the significant roles of metabolites has led to the establishment of metabolomics as an emerging methodology. Taking a systemic approach, metabolomics allows to simultaneously quantify large cohorts of metabolites in a given sample^8^. Metabolomics analysis includes two complementary approaches. Untargeted metabolomics methods identify changes in the metabolites and their relative differences between samples, yet the lack of quality control measures, such as internal standards and calibration curves, often results in reduced accuracy. In parallel, targeted metabolomics provides an accurate account of the levels of the tested metabolites, but is limited to a pre-determined set of molecules. The combination of both methodologies in iterative cycles allows confident quantitation of the relevant metabolites, thereby facilitating both discovery and hypothesis-driven research^9^. Semi-targeted methods have therefore been developed to combine the advantages of both approaches. These methodologies have provided comprehensive data sets describing the levels of numerous metabolites under various physiological and pathological conditions. Metabolomics increasingly employs a data science approach due to the vast amount of data acquired in each individual experiment and the total volume of high-quality biological and medical data. Metabolite abundances directly reflect biological perturbations originating from collective modifications of the genome, transcriptome, and proteome. Moreover, many of the most common human diseases involve disrupted metabolism at the level of the organism and/or the individual cell, bearing a clear effect on metabolites level. As a result, metabolomics is extensively utilized to develop precision medicine applications, providing specific diagnosis and personalized treatment tools for various pathological conditions characterized by phenotypic heterogeneity, including dermatomyositis, pulmonary vascular disease, and liver pathologies^10–12^. However, in spite of this vast amount of experimental findings, the role of each metabolite under a given set of conditions is far from being understood. The next challenge of the metabolomics field would therefore be to go beyond the simplistic lists of metabolites to deciphering their functions. This leap will provide insights into the basic mechanisms underlying diverse types of disease conditions, thereby advancing future efforts for developing novel therapeutics.

**Fig. 1:**
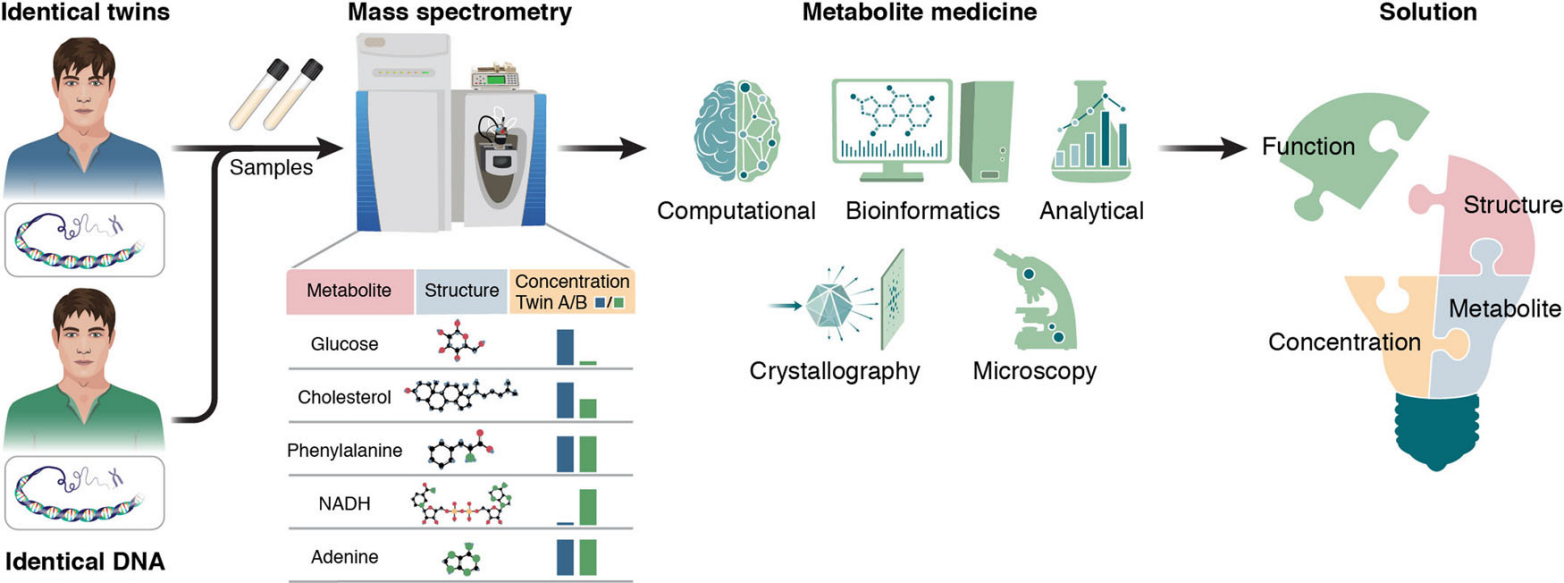
Metabolite medicine. Standard metabolomics methodologies allow to differentiate between samples, even given an identical genome, as in the displayed example. Yet, the output of such methodologies, namely the levels of diverse metabolites in each sample, requires further analysis to decipher the function of each such compound. Moreover, a standardized database that gathers high volume metabolomics data is currently not available, thus hindering the translation of individual experiments to more inclusive conclusions. The metabolite medicine concept aims to bridge this gap, allowing to obtain further information about each candidate molecule before employing biological model systems. The framework includes computational and bioinformatic analysis, the execution of which requires the establishment of an inclusive database, allowing to connect metabolites to diverse related pathologies. In parallel, the chemical composition of each compound, which dictates possible intrinsic properties, as well as its aggregation propensity and the formation of various assemblies, are to be analyzed using crystallography, microscopy, spectroscopy, and other in vitro methods. Taken together, these data can provide a more accurate representation of the metabolites involved in the studied conditions, thereby setting the basis for more focused and effective biological and clinical experiments to test and verify the metabolite functions. Moreover, this suggested infrastructure aims to aid in the identification process of multiple metabolomics analyses through the integration of structural, chemical and pathological data on each compound under a given investigated condition, thus providing an indication of its possible annotation.

Here, we present a novel concept, designated metabolite medicine, aimed to provide a conceptual framework for the functional analysis of metabolites in pathological conditions. In general, the goal of this concept is to facilitate not only the analysis of such complex samples, but also the exploration of possible mechanisms of action of these small molecules. Thus, after acquiring the list of differential metabolites, each should be linked to its known biological and chemical activities. This can be accomplished *via* an inclusive infrastructure that implements analytical, microscopy and crystallographic techniques together with bioinformatics and computational methodologies (Fig. 1). As outlined herein, diverse in vitro analyses can be utilized to gain insight into the chemical and structural properties of each candidate metabolite. In parallel, thorough computational analysis aimed to identify metabolites associated with diverse pathologies still requires the establishment of an inclusive database. Subsequently, the most promising candidate metabolites can be tested in biological and clinical experiments, or *via* integrated omics, as shown in multiple functional metabolomics studies^13,14^. Thus, the metabolite medicine approach can be implemented to further refine metabolomics data, thereby reducing time-consuming utilization of biological and clinical models.

## Metabolite medicine

As mentioned, the first step of the analysis focuses on the generation of such a metabolites list using both the targeted and the untargeted approach. Due to the complex and dynamic nature of the metabolome, a combination of highly sensitive and accurate analytical platforms is required to cover the full spectrum of metabolites in different organisms, tissues or fluids. The most commonly employed method is mass spectrometry (MS), which offers quantitative analysis of metabolites with unparalleled sensitivity and selectivity. The use of a tandem mass spectrometer (MS/MS) provides highly sensitive data and structural information, allowing to better decipher given compounds. Moreover, to overcome sample complexity and allow for metabolite separation prior to detection, the coupling of liquid chromatography (LC) to MS/MS (LC-MS/MS) is required to facilitate metabolite identification and quantitation^15,16^. Yet, chemical annotation, namely the identification of as many compounds as possible, is still a key challenge in untargeted metabolomics^9^.

Metabolomics research aims to measure and identify both endogenous and exogenous small molecules in a given sample. The metabolomics analysis of cells, tissues, and body fluids reflects the biochemical status of the sample and may serve as a direct readout of the current phenotype, either normal or pathological, of the organism. While different molecular techniques can identify changes at the genetic level, it remains unclear how mutations, single nucleotide polymorphisms or other changes in the DNA or in gene expression are manifested in the cell phenotype. Metabolism and biochemical reactions, on the other hand, receive inputs from every level of cellular regulation and thus better represent the actual cellular output. For example, in the case of identical twins who have the same genomic background, metabolomics might predict the tendency of one to develop a certain disease, thus providing valuable information. In this regard, metabolomics has been successfully used to provide insights into the course of diverse diseases, discover novel biomarkers, and shed light on the impact of drug metabolism and drug effects in vivo. While obtaining the list of metabolites of each analyzed sample is one challenge, understanding the basis for their change in a specific situation and exploring their functional role in the pathological mechanism might be an even greater one. As mentioned above, metabolites participate in various biological and chemical activities in the body including catabolic and anabolic pathways, small molecule regulators, and active biomolecules that regulate important cellular and physiological processes, designated as epimetabolites^17^. Moreover, recently it was shown that metabolites have the ability to self-assemble and form supramolecular fibrillar amyloid structures which could provide another dimension for the epimetabolomic phenomenon. These assemblies, termed metabolite amyloids, were shown to be pathologically related to several inherited metabolic disorders. The diverse set of capabilities presented by these small molecules potentially suggests their multiple functional roles in a certain disease including those related to abnormal proteostasis conditions^18,19^. In light of this, when trying to decipher the connection between the differential metabolites and their mechanism of action in a given pathology, their link to other pathologies with similar symptoms, physical localization in the body based on high-resolution microscopy, propensity to aggregate and form a variety of assemblies and their chemical structure derived from crystallographic analyses should all be considered. The ability to integrate data regarding a specific metabolite in terms of its chemical structure as well as its native and pathological function may lead to a better understanding of its mechanism of action in an unexplored system, thereby facilitating a more focused and specifically-targeted functional metabolomics research^13,14^.

One example that reflects the metabolite medicine concept is the discovery of the mechanistic role of phenylalanine amyloid assemblies in phenylketonuria (PKU) pathology^3^. For decades, phenylalanine was known to accumulate in PKU, one of the most common IEM disorders. While phenylalanine was utilized as a hallmark biomarker for the diagnosis of PKU, for example in newborn metabolomics screening, its pathological mode of action was not fully understood^20,21^. Interestingly, previous work that examined the amyloid aggregation propensity of all 20 coded amino acids revealed that phenylalanine, as an isolated amino acid, has one of the highest aggregative propensities^22^. Moreover, the diphenylalanine dipeptide, the core recognition motif within the β‐amyloid polypeptide, was found to form well-ordered amyloid assemblies, showing optical and functional properties characteristic of protein and poly-peptide amyloids^23^. Together with the aromatic nature of phenylalanine, which is known to play a significant role in amyloid aggregation^24^, this line of evidence proposed a possible functionality of the amino acid as an amyloidogenic building block. Indeed, as initially discovered by us^2,3^ and followed by others^4,25,26^, phenylalanine possesses the ability to self-assemble and form supramolecular nano-fibrils, which exhibit typical ultrastructural, biophysical and biochemical properties. Moreover, we have shown these phenylalanine assemblies to confer a cytotoxic effect via an apoptotic cell death mechanism, which could be rescued using specific antibodies raised against these fibrillar species. The presence of anti-phenylalanine fibrils antibodies in a PKU mouse model, as well as the presence of these fibrils in post-mortem brain samples of individuals affected with the disease, potentially suggest a role for the phenylalanine assemblies in the etiology of PKU^2,3^. To study whether these observations represent a general amyloid-like mechanism prevalent in other IEM disorders, we tested additional metabolites that accumulate in IEM disorders. We revealed that several other metabolites could self-assemble to form ordered amyloid-like ultrastructures in solution with an array of characteristics that resemble canonical proteinaceous amyloids. We established a new terminology, designated *metabolite amyloid*, describing the formation of amyloid-like assemblies by metabolites, which implies a general phenomenon of amyloid formation, not limited to proteins and peptides^2,27–31^. Interestingly, specific IEM disorders present a neuropathology and a series of neurological symptoms similar to those observed in neurodegenerative disorders^32,33^, in which the formation of amyloids and their pathological role are well established. These symptoms might therefore be triggered by the presence of metabolite amyloid assemblies. On top of the excess amount of a certain metabolite in a specific pathology, this example highlights the importance of thoroughly investigating the functionality of metabolites by taking into account their chemical composition, which dictates possible intrinsic properties, as well as their appearance in other known diseases.

## Metaboloinformatics

**Fig. 2:**
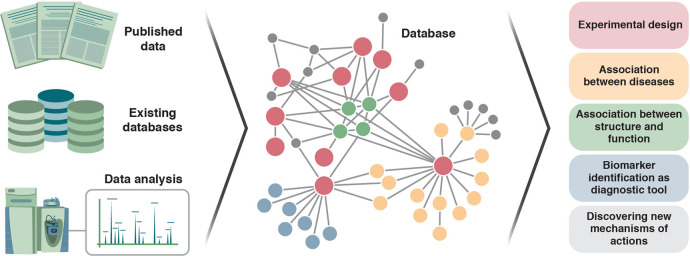
Metaboloinformatics. Establishment of an inclusive database that connects between metabolites and their related pathologies would allow a variety of applications, including improved experimental design and functional analysis.

Similar to the development of the bioinformatics interdisciplinary field, which combines technologies from computer science and statistics to analyze biological data, the metaboloinformatics sub-field should be discussed. This approach aims to investigate a diverse set of questions from a computational perspective and assimilate methods which facilitate a deeper understanding of data^34^. However, as presented here, we view metaboloinformatics as a much broader approach. Establishing a database of metabolites linked to their related pathologies as well as their possible known functions as druggable small molecules might aid to provide a significantly more comprehensive tool (Fig. 2). Moreover, the development of automated scripts for the data processing pipelines, as well as tools that will improve future experimental design and statistical analysis, is well required. While thousands of small molecules are detected in a single metabolomics experiment, most of these molecular entities remain unknown features that are hard to identify. Thus, one of the major challenges in untargeted metabolomics studies is to match as many of these unidentified molecules to their chemical structure or formula^9^. The correct chemical annotation is essential to gain insights into the involvement of the metabolites in the investigated pathology. In order to improve the annotation or even identification of metabolites in a given experiment, inter-lab collaboration and establishment of comprehensive databases, which will gather all known data on metabolites as being pathologically associated, are required.

Genomics research has made an important leap from the mere sequencing of an extensive collection of full genomes to understanding the precise role of the identified genes and the interactions of their products (both RNA and proteins). Similarly, the field of metabolomics is on the verge of shifting from the collection of enormous amounts of data regarding the levels of metabolites under various conditions to deciphering the function of each metabolite in a given experimental system. Nevertheless, while the genomic information is predetermined, the amount of the metabolic data is practically infinite and thus requires advanced and creative metaboloinformatics analysis. By combining experimental and bioinformatical tools, the metabolite medicine model described here suggests a methodological framework for advancing such studies, thereby allowing to gain further insights into the unexplored roles of metabolites.
